# Association of elevated autoantibody to high expression of GNAS in hepatocellular carcinoma

**DOI:** 10.1016/j.heliyon.2023.e22627

**Published:** 2023-11-20

**Authors:** Keyan Wang, Cuipeng Qiu, Mengtao Xing, Miao Li, Bofei Wang, Hua Ye, Jianxiang Shi, Liping Dai, Xiao Wang, Peng Wang

**Affiliations:** a Henan Institute of Medical and Pharmaceutical Sciences, Zhengzhou University, Zhengzhou, 450052, Henan, China; b State Key Laboratory of Esophageal Cancer Prevention & Treatment, Zhengzhou University, Zhengzhou, China; c Department of Epidemiology and Health Statistics & Henan Key Laboratory for Tumor Epidemiology, College of Public Health, Zhengzhou University, Zhengzhou 450001, Henan, China; d Department of Pharmacology, China Pharmaceutical University, Nanjing 210009, China

**Keywords:** HCC, Detection, Autoantibody, Protein expression, Gene mutation

## Abstract

**Purpose:**

This study was based on hepatocellular carcinoma (HCC) patients of early-stage to explore the diagnostic capability and possible production causes of anti-GNAS autoantibody.

**Methods:**

We evaluated the frequency of anti-GNAS autoantibody in sera from patients with early-stage HCC by enzyme-linked immunosorbent assay (ELISA) and the expression of GNAS protein in early-stage HCC tissues by immunohistochemistry. Western blotting (WB) and real-time polymerase chain reaction (RT-PCR) were utilized to examine the expressions of GNAS protein and mRNA in cell lines. GEO and International Cancer Genome Consortium (ICGC) databases were inquired to explore mRNA expression and mutation of GNAS in HCC tissues.

**Results:**

The positive rates of anti-GNAS autoantibody in HCC patients at clinical stage I (78.1 %) and clinical stage II (57.1 %) were all significantly higher than that in healthy control (20 %). There was also a significant difference in GNAS protein expression between HCC and its adjacent normal liver tissues. The results from WB and RT-PCR showed a significant difference at the mRNA level but no statistical difference at the protein level between HCC and normal liver cell lines. The difference in mRNA level between HCC and adjacent normal liver tissues was verified to be significant. Furthermore, the ICGC database demonstrated a 10.6 % mutation frequency for GNAS in HCC patients.

**Conclusion:**

The coordination of elevated anti-GNAS autoantibody, high expression of GNAS in the mRNA and protein levels in HCC, and high frequency of GNAS mutation indicates that anti-GNAS autoantibody may be used as an early indicator of HCC.

## List of abbreviations

HCC: hepatocellular carcinoma
GNAS: guanine nucleotide-binding protein alpha subunit
GPCR: G protein-coupled receptor
ELISA: enzyme-linked immunosorbent assay
WB: western blotting
RT-PCR: real-time polymerase chain reaction
ICGC: International Cancer Genome Consortium
AFP: alpha-fetoprotein
TAAs: tumor-associated antigens
SDS-PAGE: sodium dodecyl sulfate-polyacrylamide gel electrophoresis
GAPDH: glyceraldehyde-3-phosphate dehydrogenase

## Introduction

Hepatocellular carcinoma (HCC) is one of the most common malignant tumors worldwide and is especially prevalent in Africa and Asia. However, HCC usually occurs without any warning symptoms, and most patients are found at a late stage when no effective therapy can be taken, leading to poor prognosis. Alpha-fetoprotein (AFP), an effective serological marker, is widely used in the clinical detection and diagnosis of HCC but is limited due to its low sensitivity and specificity [1]. Thus, it is vital to identify effective and novel candidate biomarkers to be used for early detection of HCC.

Exploratory research on autoantibodies against tumor-associated antigens (TAAs) as biomarkers for cancer has been ongoing for many years [[2], [3], [4], [5]]. Evidence indicates that autoantibodies precede clinical confirmation of a tumor by several months or years [6], which is the basis for their use as early indicators for cancer. Compared to other serological biomarkers, such as circulating cell-free DNA, TAAs, or RNA, autoantibodies enlarged by the immune system are more stable and durable, and they have higher titers in the serum despite lower levels of the corresponding antigen, which enables them to be ideal noninvasive biomarkers [7].

Autoantibody to GNAS was elevated in pre-HCC patients in previous studies [8,9]. GNAS functions as a transducer in numerous signaling pathways controlled by G protein-coupled receptors (GPCRs) [10], and the activation of GPCRs can stimulate different G protein-dependent and independent pathways, leading to changes in gene transcription, cell survival and motility, and normal and malignant cell growth [11]. GNAS is one of the most frequently mutated genes in human cancer and its mutations are widespread in various malignancies [[12], [13], [14], [15], [16], [17]]. The activating mutations of GNAS were detected in approximately two-thirds of intraductal papillary mucinous neoplasms (IP-MNs) of the pancreas [18] and was reported to cooperate with the inactivation of APC and contribute to colorectal tumorigenesis [19]. Nault's study in HCC cell lines demonstrated that mutations in GNAS induce inflammation and Stat3 activation, thereby enhancing the role of Stat3 activation in liver tumorigenesis [20]. These studies suggest that GNAS are involved in the formation of tumors in various tissues and organs through gene mutation and various signal pathways. Therefore, it is necessary to explore the GNAS gene/protein and its corresponding autoantibody along with the correlation between them in HCC patients.

Even though the underlying causes of autoantibody production remain to be elucidated, it is believed that autoantibody may be generated by the immune system in response to mutated proteins, aberrantly expressed proteins or post-translationally modified proteins [21]. Previous studies [8,9] found that anti-GNAS autoantibody was elevated in HCC patients but there was no exploration on GNAS protein/mRNA expression and GNAS mutation which may cause autoantibodies to increase in early HCC patients. Herein, we expanded our previous study to mRNA and protein levels even gene mutation, and used early-stage HCC patients to explore the autoantibody response to GNAS mutation and its protein/mRNA expressions.

## Materials and methods

### Serum samples

In the current study, 125 serum samples from HCC patients at clinical stages I-II and 125 serum samples from healthy controls were obtained from the serum bank of the Tumor Epidemiology Laboratory of Zhengzhou University (Henan, China). HCC patients were diagnosed according to the established criteria in 2019 in China [22]. Based on the Chinese staging guideline for liver cancer [23], 41 out of 125 (32.8 %) patients were in clinical stage I, 84 out of 125 (67.2 %) patients were in stage II. HCC patients at stage I–II were defined as early-stage HCC patients. No HCC patients received chemotherapy or radiation treatments before the sera were collected and all healthy individuals had no history of liver diseases. This study was performed in accordance with the rules of the Declaration of Helsinki, which was approved by the Ethics Committee of Zhengzhou University (ZZURIB 2019-001) and all the subjects had signed informed consent. The clinical characteristics of the participants are demonstrated in Table 1.

**Table 1:** Characteristics of participants.

Variables	HCC	Healthy
Number	125	125
Gender, n (%)		
Male	103 (82.4)	102 (81.6)
Female	22 (17.6)	23 (18.4)
Age, years		
Mean ± SD	55.8 ± 10.9	57.7 ± 10.8
Range	32.0–87.0	35.0–87.5
TNM, n (%)		
I	41 (32.8)	
II	84 (67.2)	
AFP, ng/ml		
Median (P25, P75)	32.4 (5.9, 987.0)	3.8 (3.3, 4.9)
≥20, n (%)	59 (47.2)	0 (0)
<20, n (%)	47 (37.6)	90 (72.0)
NA, n (%)	19 (15.2)	35 (28.0)

HCC, hepatocellular carcinoma; SD, standard deviation; P25, 25 % percentile; P75, 75 % percentile; AFP, alpha-feto-protein; NA, not available.

### Enzyme-linked immunosorbent assay (ELISA)

ELISA was used to detect anti-GNAS autoantibody in serum samples. Detailed procedures for ELISA were described in our previous study [24]. In brief, purified GNAS recombinant protein was purchased from Cloud-Clone Corporation (Wuhan, China), and it was diluted at 0.5 μg/ml and coated onto a 96-well microliter plate. Sera were diluted at 1:100. Horseradish Peroxidase-conjugated Goat anti-human IgG was used as a secondary antibody at 1:10000 dilution. The substrate was a mixture of 50 % 3, 3′ 5, 5′-Tetramethybenzidine (TMB) and 50 % Hydrogen Peroxide. The reaction was stopped with a stopping solution (2 M H2SO4). The absorbance value of each well was read at 450 nm and 620 nm on a microplate reader (Thermo Fisher Scientific). In addition, 8 fixed human serum samples and 2 blank controls were set up on each 96-well plate for normalization of absorbance value from different plates and the adjustment of background in each individual plate, respectively.

### Immunohistochemistry (IHC) with tissue microarray

The clinical tissue microarray (HLivH180Su07) was purchased from Xinchao Biotech (Shanghai, China) and consisted of 61 HCC tissues and 61 adjacent normal liver tissues from early HCC patients. IHC assay was performed by using mouse monoclonal antibody against GNAS (Proteintech, China, 1:100 dilution) according to the manufacturer's recommendations to analyze GNAS protein expression level. The results were read by two independent pathologists. The intensity and stained area were counted in the IHC scoring process [25]. The staining intensity was scored as 0 (negative), 1 (weak), 2 (moderate), and 3 (strong). The percentage of staining positive cells was scored as 1 (up to 25 %), 2 (26–50 %), 3 (51–75 %), and 4 (76–100 %). The final IHC scores were calculated by multiplying the intensity by the scores corresponding to the percentage of positive cells. The positive rate of GNAS detection was counted based on the IHC score ≥8 (95 % quantile of IHC score of paracancerous tissues).

### mRNA level detection in HCC cell lines

L-02 and SNU-449 cell lines were cultured in RPMI-1640 Medium (Gibco, Massachusetts, USA) supplemented with 10 % fetal bovine sera (Gibco, Massachusetts, USA). Hep3b was cultured in Modified Eagle Medium (Gibco, Massachusetts, USA) with 10 % fetal bovine sera. HepG2 was cultured in Dulbecco's Modified Eagle Medium (Gibco, Massachusetts, USA) with 10 % fetal bovine sera. Cells were maintained in an incubator with 5 % CO2 at 37 °C with the initial seeding density of 3 × 10^5^ each well in 6-well plates. When the cells' fusion rate reached more than 90 %, the total RNA in each cell lines was extracted using TRIzol reagent (TaKaRa, Kyoto, Japan) according to the manufacturer's instructions. The primer for GNAS was designed based on the reference mRNA sequence in GenBank (NM-006496) and ordered from Sango Biotech company, and the sequences were as follows: GAPDH-Forword: CAGGAGGCATTGCTGATGAT; GAPDH-Reverse: GAAGGCTGGGGCTCATTT; GNAS-Forword: GCCTGCTACGAACGCTCCAAC; GNAS-Reverse: TCCTGATCGCTCGGCACATAGTC. The extracted RNA was used for complementary DNA synthesis with the Reverse transcription kit (Thermo fisher scientific, Massachusetts, USA). Real-time polymerase chain reaction (RT-PCR) was carried out on QuantStudio 3 (Thermo fisher scientific, USA). GAPDH gene was used as an endogenous control and the relative level of GNAS mRNA was calculated using the 2^-△△Ct^ method.

### Western blot analysis

Cell lysates from human HCC cells (SNU449, HepG2, and Hep3b) and human fetal liver cells (L02) were electrophoresed with 12 % SDS-PAGE Gel and transferred onto Nitrocellulose (NC) membrane. The membranes were blocked with 5 % non-fat milk in Tris-buffered saline with 0.05 % Tween20 (TBST) (pH = 7.4) at 4 °C overnight and then incubated for 1 h at room temperature with 1:1500 dilution of mouse monoclonal anti-GNAS (ProteinTech., China), as well as 1:1000 dilution of rabbit monoclonal anti-GAPDH (Cell Signaling Tech., USA). HRP-conjugated goat anti-mouse IgG at 1:3000 (BD Biosciences, USA) and anti-rabbit IgG at 1:2000 (BD Biosciences, USA) was used as secondary antibodies. The signals of immunoreactive bands were detected with an enhanced chemiluminescence kit (Millipore Corporation, USA) on Azure Biosystems C300 (USA).

### Bioinformatics analysis

In order to learn more about the expression and mutation of GNAS in HCC patients, we resorted to bioinformatics methods and techniques for data acquisition and analysis. Gene Expression Omnibus (GEO) dataset was investigated for the expression of GNAS at the mRNA level in HCC tissues. GNAS mutation in HCC tissues across 5 research cohorts was explored in International Cancer Genome Consortium (ICGC, https://dcc.icgc.org/, April 10, 2022). Human Protein Atlas (HPA, https://proteinatlas.org, May 15, 2021) was consulted to determine the expression of GNAS protein in HCC tissues.

### Statistical analysis

IBM SPSS (Version 21.0, Chicago, IL) and GraphPad Prism (Version 6.0, La Jolla, CA, USA) were utilized in the study. The Mann-Whitney *U* test and Kruskal-Wallis test were applied to compare the difference in the levels of quantitative variables, while the Pearson Chi-Square test and Fisher's Exact test were performed to compare the differences in the frequency of categorical variables. The Wilcoxon paired-samples signed rank test was used to analyze the difference in IHC score between HCC tissue and the adjacent normal liver tissues. Receiver operating characteristic (ROC) curves were generated and area under the ROC curve (AUC) with sensitivity and specificity together were used to evaluate the diagnostic value. The cut-off value of anti-GNAS autoantibody was defined at the point of the maximum Youden index when the specificity was 80.0 % according to the analysis of the ROC.

## Results

### Study design

**Fig. 1:**
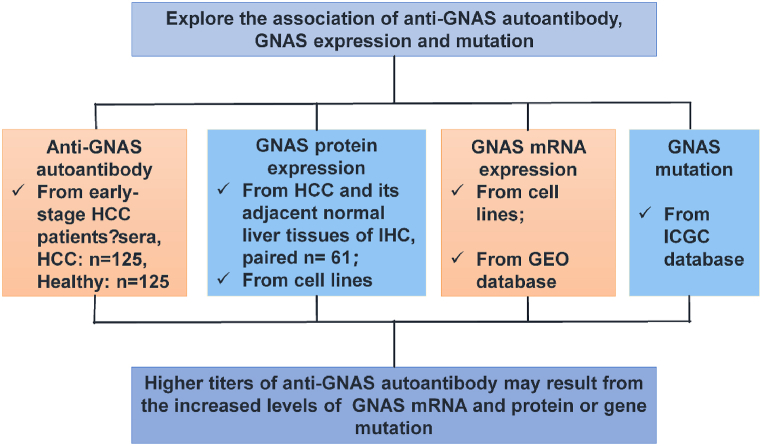
**Workflow chart of the study.** First, 125 early-stage HCC patients and 125 healthy controls were recruited to evaluate the potential value of anti-GNAS autoantibody for early detection of HCC by ELISA. GNAS protein expressions in 61 paired HCC or adjacent normal tissues were tested by IHC, subsequently, the levels of GNAS protein in three HCC cell lines and the normal liver cell line were investigated by Western blot. GEO datasets were inquired to explore the mRNA expression level of GNAS, meanwhile, the mRNA levels in three HCC cell lines and the normal liver cell line were detected by real-time PCR. Additionally, ICGC Data Portal was also queried for exploring the mutation frequency of *GNAS* in HCC tissues.

As shown in Fig. 1, 125 early-stage HCC patients and 125 healthy controls matched in age and gender (*P* > 0.05 for both) were recruited to evaluate the potential value of anti-GNAS autoantibody in the early detection of HCC by ELISA. GNAS protein expressions in 61 paired HCC or adjacent normal tissues were tested by IHC, Subsequently, the levels of GNAS protein in three HCC cell lines and the normal liver cell line were evaluated by Western blot. The GEO dataset was used to explore the mRNA expression level of GNAS, and the mRNA levels in three HCC cell lines and the normal liver cell line were detected by RT-PCR. Additionally, ICGC Data Portal was also queried for exploring the mutation frequency of *GNAS* in HCC tissues.

### Level and frequency of anti-GNAS autoantibody in sera of patients with early-stage HCC

**Fig. 2:**
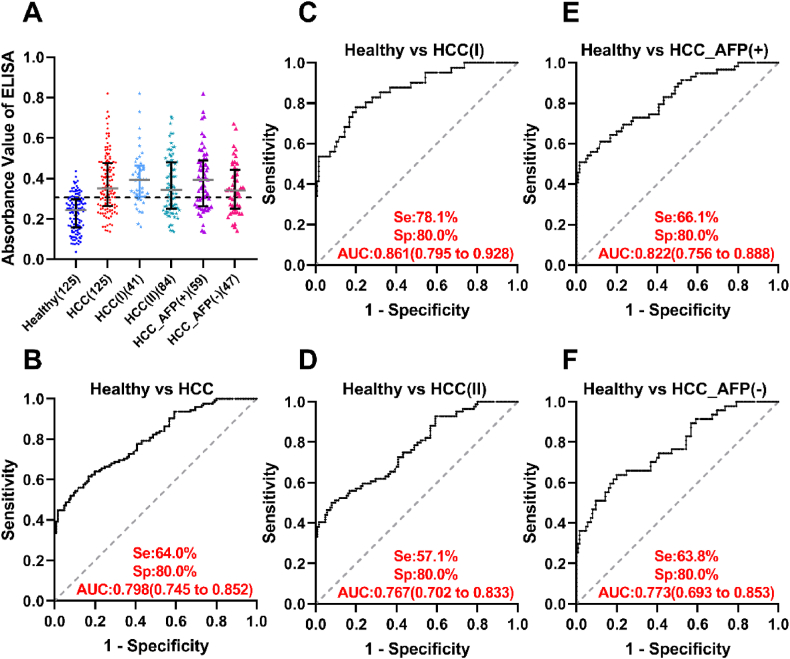
**The scatter plot of autoantibody to GNAS in different groups (A), and ROC curves of autoantibody to GNAS when distinguishing early HCC and different subgroups of HCC from healthy control (B, C, D, E, F).**HCC, hepatocellular carcinoma; AFP, alpha-feto-protein; ELISA, Enzyme-linked immunosorbent assay; HCC(I), HCC patients of TNM stage I; HCC(II), HCC patients of TNM stage II; HCC_AFP (+), HCC patients of AFP value greater than or equal to 20 ng/mL; HCC_AFP (−), HCC patients of AFP value less than 20 ng/mL; Se, sensitivity; Sp, specificity; AUC, area under the receiver operating characteristic curve; 95 % confidence interval of AUC in brackets.

This study mainly focuses on subjects with early-stage HCC (Fig. 1). The validation of anti-GNAS autoantibody was performed on a cohort consisting of 125 early-stage HCC patients and 125 healthy controls that were matched by age and gender. The characteristics of patients and healthy controls are shown in Table 1. In this dataset, the level of anti-GNAS autoantibody in early HCC sera was higher than that in healthy controls (*P* < 0.05), as illustrated in Fig. 2A. Fig. 2B and Table 2 showed that the autoantibody to GNAS with an AUC of 0.798 can differentiate 64.0 % of early-stage HCC patients from healthy controls while the cut-off value designating positive reaction was set as the corresponding points to the largest Yueden's index when the specificity reached 80 %.

**Table 2:** The levels and frequency of autoantibody to GNAS evaluated by ELISA.

Categories	N	absorbance value, P50 (P25, P75)	P*	Positive rate, n (%)	P^#^
HCC vs Healthy			<0.001		<0.001
HCC	125	0.351 (0.263, 0.476)		80 (64.0)	
Healthy	125	0.243 (0.157, 0.297)		25 (20.0)	
HCC subgroups					
Gender			0.378		0.347
Male	103	0.351 (0.261, 0.474)		64 (62.1)	
Female	22	0.353 (0.300, 0.486)		16 (72.7)	
AFP			0.343		0.810
≥20 ng/mL	59	0.393 (0.262, 0.490)		39 (66.1)	
<20 ng/mL	47	0.341 (0.250, 0.443)		30 (63.8)	
NA	19	0.375 (0.269,0.482)		11 (57.9)	
Age			0.022		0.022
≥55 years	67	0.404 (0.289, 0.482)		49 (73.1)	
<55 years	58	0.333 (0.245, 0.434)		31 (53.4)	
TNM stage			0.187		0.022
I	41	0.393 (0.310, 0.464)		32 (78.1)	
II	84	0.344 (0.250, 0.480)		48 (57.1)	
HBV status			0.038		0.121
Yes	92	0.338 (0.254, 0.451)^a^		56 (60.9)	
No	26	0.432 (0.263, 0.527)^a,b^		17 (65.4)	
NA	7	0.444 (0.354, 0.671)^b^		7 (100)	

HCC, hepatocellular carcinoma; AFP, alpha-feto-protein; NA, not available; N, number; P50, 50 % percentile; P25, 25 % percentile; P75, 75 % percentile; Positive rate, the OD value of ELISA greater than or equal to 0.305 (cutoff value); P*, the results of Mann-Whitney *U* test or Kruskal-Wallis test, if the letters in the upper right corner of “HBV status” (a, b, c) between two subgroups are totally different, the difference between the two groups is considered to be significant (*P* < 0.05); P^#^, the results from Pearson Chi-Square test or Fisher's Exact test.

As shown in Fig. 2 and Table 2, the performance of anti-GNAS autoantibody was further explored in each of the HCC subgroups such as clinical stages, AFP levels, gender, age, and HBV status. The results demonstrated that the positive rate (78.1 %) and AUC (0.861) in clinical stage I HCC patients were significantly higher than those (57.1 % and 0.767) in clinical stage II HCC patients (Pearson Chi-Square test, *P* = 0.022; De Long's test for AUC, *z* = 1.975, *P* = 0.048), as shown in Fig. 2C and D. There was no statistical difference in AUCs between AFP (−) group and AFP (+) group (De Long's test, *z* = 0.928, *P* = 0.354) (Fig. 2E and F). In addition, the level and frequency of anti-GNAS autoantibody were significantly higher in early HCC patients with older age (≥55 years old) than younger patients (<55 years old). There were no statistical differences across HBV status, gender, and AFP levels.

### Expressions of GNAS protein in human HCC tissues and cell lines

**Fig. 3:**
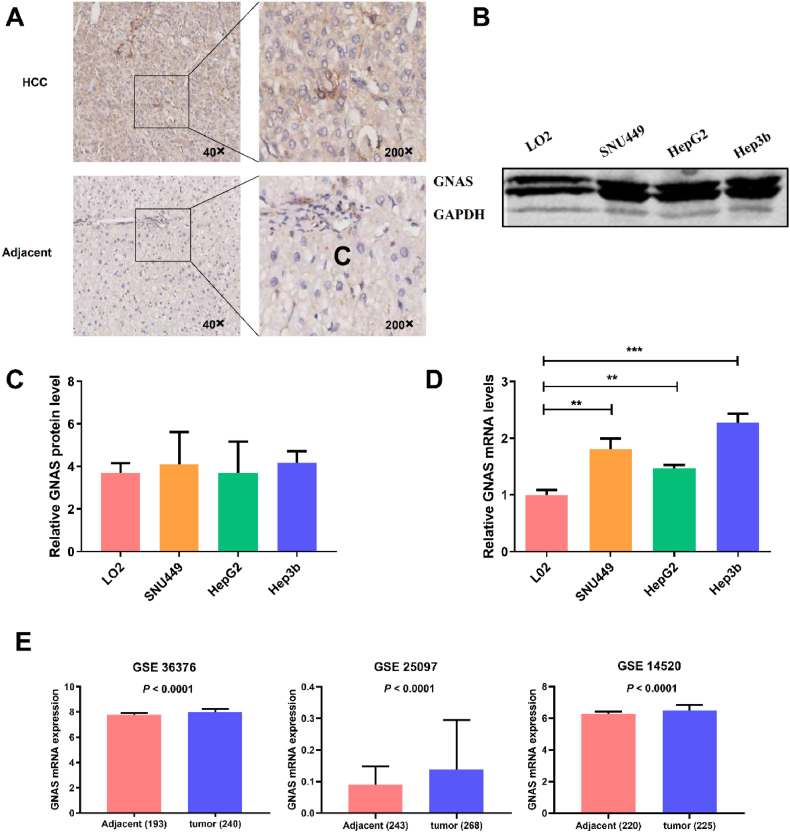
**the expression of GNAS in tissue and cell lines.** (A) Immunohistochemical staining of GNAS in HCC tissue and adjacent normal tissue slides. Positive stain pattern of GNAS in representative HCC tissue of TNM stage I, weak stain pattern of GNAS in the representative adjacent normal tissue of TNM stage I (40× and 200 × magnifications) (B) the cropped gel displaying protein levels of GNAS tested by western blotting in cell lines. The lanes of upper lines from left to right showed the GNAS protein levels in cell lines of L02, SNU449, HepG2, and Hep3b, and the following lanes were the expression levels of reference protein GADPH. The corresponding uncropped full-length gel can be found in Additional file 1. (C) the relative expression of GNAS to GAPDH in protein level from a normal liver cell line (L02) and three HCC cell lines (SUN449, HepG2, and Hep3b). (D) the relative expression of GNAS to GAPDH in mRNA level from normal liver cell line (L02) and three HCC cell lines (SUN449, HepG2 and Hep3b), **, *P* < 0.01, ***, *P* < 0.001. (E) the expressions of GNAS at the mRNA level from three datasets of the GEO database, numbers in horizontal-axis parentheses represent the sample size.

To understand GNAS expression at the protein level in HCC tissues, we queried the Human Protein Atlas (HPA; https://www.proteinatlas.org/) and found that GNAS protein expression data was antibody-based without simultaneous contrast between HCC tissues and normal or paracancerous liver tissues. As a result, IHC experiment was performed in an HCC tissue microarray including 61 HCC tissues and 61 paracancerous tissues which were from early-stage HCC patients. The results showed that GNAS protein was mainly localized to the cytoplasm and there was a significant difference in expression level reflected by the IHC score between HCC tissues and adjacent normal liver tissues (*P* < 0.05, Table 3). When the cutoff value was set as 8 of the IHC score, the GNAS protein revealed a statistically higher positive rate in HCC tissues than in adjacent normal liver tissue (*P* < 0.05), the IHC results for a pair of representative HCC and paracancerous tissues are shown in Fig. 3A. When we analyzed the correlations of GNAS expression in HCC tissues and the clinical features, it was found that there was no statistically significant difference of GNAS protein expression in tissues across tumor sizes, pathological grades, survival periods, with or without recurrence, cirrhotic nodules, and HBV status (*P* > 0.05) (see Table 3).

**Table 3:** the expression of GNAS evaluated by IHC.

Categories	N	IHC score, Mean	P*	Positive rate, n (%)	P^#^
Cancer vs Normal			**0.030**^**a**^		**0.013**^**b**^
HCC tissue	61	7.10		44（72.1）	
Adjacent normal tissue	61	6.30		36（59.0）	
Pathological grade			0.250		0.157
I	1	4.0		0（0）	
II	37	7.38		29（78.4）	
III	23	6.78		15（65.2）	
AFP, ng/mL			0.752		0.525
≥20	43	7.26		30（69.8）	
<20	18	6.72		14（77.8）	
Tumor size, cm			0.932		0.763
≥5	27	7.11		20（74.1）	
<5	34	7.09		24（70.6）	
Recurrence status			0.772		0.493
Yes	33	7.15		25（75.8）	
No	28	7.04		19（67.9）	
Survival time, years			0.289		0.379
≥4	37	7.46		28（75.7）	
2–4	9	5.89		5（55.6）	
<2	14	7.14		11（78.6）	
Cirrhosis status			0.925		1.000
Yes	54	7.06		39（72.2）	
No	7	7.43		5（71.4）	
HBV status			0.578		0.483
Yes	59	7.14		43（72.9）	
No	2	6.00		1（50.0）	

AFP, alpha-feto-protein; HBV, hepatitis B virus; N, number; IHC: Immunohistochemistry; Positive rate: the score of IHC greater than or equal to 8; P*, the results of Mann-Whitney *U* test or Kruskal-Wallis test; P^#,^ the results of Pearson Chi-Square test or Fisher's Exact test; ^a^, resulted from Wilcoxon paired-samples signed rank test;^b^, resulted from McNemar's test.

Western blotting was performed to examine GNAS protein expression in HCC cell lines (SNU449, HepG2, and Hep3b) and normal liver cell line (L02). It was found that the GNAS protein expression level in HCC cells was slightly higher than that in normal liver cells but without statistical significance (Fig. 3B and C). The uncropped versions of Fig. 3B can be found in Supplement Fig. 1.

### GNAS mRNA expressions in HCC cell lines and tissues

We examined the mRNA level of GNAS in HCC cell lines (SNU449, HepG2, and Hep3b) and normal liver cell line (L02) by RT-PCR, and the results showed that it was significantly higher in HCC cells than that in normal liver cells (Fig. 3D).

The expression of GNAS mRNA in liver cancer tissues and adjacent non-cancerous liver tissues was investigated from GEO databases in which three datasets with large size of specimens all showed that the level of GNAS mRNA expression in liver cancer tissues is significantly higher than that in adjacent non-cancer liver tissues (Fig. 3E).

### GNAS mutations in human HCC tissues

To understand the presence of *GNAS* mutations in HCC patients and their possible contribution to the production of anti-GNAS autoantibodies, we further investigated the ICGC Data Portal. We outlined a table of *GNAS* mutations in 1670 HCC tissues across five independent cohorts not only to screen HCC tissues for *GNAS* mutations but also to measure the number of mutations in *GNAS* in each of the mutated HCC tissues. As shown in Table 4, 177 of 1670 donors were affected by 308 mutations with mutation frequency in *GNAS* ranging from 3.0 % to 21.7 % with an average of 10.6 % across the five cohorts.

**Table 4:** Frequency of *GNAS* Mutations in HCC across 5 cohorts.

Cohort	Tumor subtype	Frequencies, % (Donors affected/Donors)	N
1	HCC (Virus associated)	21.7 (56/258)	75
2	HCC (HBV-associated)	16.7 (67/402)	141
3	HCC (alcohol and adiposity associated)	6.7 (17/252)	53
4	HCC (Virus associated)	6.6 (26/394)	31
5	HCC	3.0 (11/364)	8

177 of 1670 donors were affected by 308 mutations across 5 cohorts. N, numbers of mutations; HCC, hepatocellular carcinoma; HBV, hepatitis B virus.

## Discussion

In recent years, autoantibodies to TAAs have become a research hotspot in the field of tumor biomarkers [26,27]. A number of studies have shown that there are high levels of anti-TAA autoantibodies in the sera of patients with HCC and these autoantibodies have the potential to diagnose HCC [27,28]. Anti-Ku86 autoantibody in HCC patients was reported showing higher sensitivity and specificity, especially for early-stage HCC patients, but with a smaller sample size [29,30]. Another marker IgGL-3 showed better diagnostic capacity than AFP, but it is a derivative of immunoglobulin G rather than a specific autoantibody; it does not have the advantages of autoantibodies such as early appearance, easy detection, and low blood consumption [31]. Autoantibody to GNAS was identified from sera of HCC patients and found to have the potential to be a biomarker for the early detection of HCC [8,9]. However, in a previous study, the anti-GNAS autoantibody level in HCC patients with early-stage tended to be higher than that in HCC patients with advanced stage without a significant difference. Although elevated anti-GNAS autoantibody was observed in other cancer patients, there was no detection and evaluation of the corresponding antigen [[32], [33], [34]].

In our current study, 125 early-stage HCC patients were used as research subjects, the level and frequency of anti-GNAS autoantibody were significantly higher in early-stage HCC patients than those in healthy controls. Moreover, in early HCC patients, the positive rate of autoantibody to GNAS at TNM stage I (78.1 %) was significantly higher than that at stage II (57.1 %). The refined distribution of this autoantibody to GNAS in early-stage HCC patients suggested that the autoantibody presents with a relatively high level in the very early stage of HCC formation. To further explore the expression of the target antigen of anti-GNAS autoantibody, IHC was performed by using an HCC tissue microarray which included 61 HCC tissues and 61 adjacent liver tissues from early-stage HCC patients. The testing results showed that the GNAS protein expression level was higher in HCC tissues compared to that in the corresponding adjacent liver tissues (P < 0.05). The consistent increase of anti-GNAS autoantibody in early-stage HCC patient sera and the GNAS protein in early-stage HCC tissues suggests that there may be a relationship between them. The high frequency of *GNAS* mutation and high expression level of GNAS mRNA were also observed in HCC tissues and HCC cell lines, respectively. Similar results have been seen in others' studies. In Kishimoto's study [35], overexpression of cysteine sulfinic acid decarboxylase (CSAD) at mRNA and protein levels was observed in the precancerous liver in rats with HCC, and the auto-antibody to CSAD was also detected in sera of the HCC-bearing rats with a much higher level than that in normal rats, proposing that the high level of CSAD autoantibody resulted from increased CSAD gene or protein expression in the liver of HCC rats. Autoantibody against a commonly mutated gene product, p53, has been found in several solid cancer types including colorectal, ovarian, lung, and breast cancer [36,37]. In Ralhan's study, a strong correlation was observed between circulating anti-p53 autoantibody and p53 alterations, including p53 mutations and protein accumulation, in esophageal cancer patients [38]. Moreover, the present level of autoantibody can be associated with the mutational load in tumoral cells [39].

Compared to the studies above, our research subjects and targets were different. We focused on early-stage HCC patients and targeted the sera and tissues from early-stage HCC patients and for the first time revealed the correlation between the elevated anti-GNAS autoantibody and the high level of its target protein in stage I HCC patients. The results suggest that elevated anti-GNAS autoantibody which was present in very early-stage HCC patients may result from the higher expression of GNAS protein in HCC tissues, thus autoantibody to GNAS has more potential to be an indicator for the early detection of HCC.

It's well known that the production of autoantibodies is the humoral immune response to aberrant protein expression, gene mutations, abnormal post-translational modifications of proteins, and so on [21,37]. *GNAS*, as a common oncogene, is involved in the pathogenesis of various cancers. Regarding the role of GNAS in the occurrence and development of cancers, numerous studies focused on the higher frequency and the driving role of *GNAS* mutations in IPMNs of the pancreas [[40], [41], [42], [43]], *GNAS* gene mutation also presented in colorectal cancer, clear cell renal cell carcinomas, small cell lung cancer, and was demonstrated to be a tumor-promoting role [[44], [45], [46]]. In our current study, the investigation of the ICGC database showed that *GNAS* mutations were also found in HCC tissues with an overall frequency of 10.6 % in 1670 HCC tissues across five cohorts. The investigation results combined with other research above suggest that the product of mutated *GNAS*, as a mutated protein, may be related to the production of autoantibody to GNAS.

In conclusion, the current study demonstrated the pathophysiological manifestations of GNAS at multiple levels in early-stage HCC patients. The results suggested that elevated autoantibody to GNAS in early HCC patients might result from the high expression of GNAS protein in early-stage HCC patient tissues, and the high frequency of *GNAS* mutation may be related to the elevation of anti-GNAS autoantibody. Based on these findings, autoantibody to GNAS could be further considered as a potential biomarker for the early detection of HCC. In future studies, the simultaneous detection of these parameters in various biomaterials from the same HCC patients may provide more informative and powerful support.

## Data availability

The public datasets analyzed in the present study are available in the Gene Expression Omnibus (GEO) dataset (GSE36376, GSE25097, GSE14520), International Cancer Genome Consortium (ICGC, https://dcc.icgc.org/, April 10, 2022) and Human Protein Atlas (HPA, https://proteinatlas.org, May 15, 2021). Other supporting materials were all included in the manuscript, or raw data can be obtained from the corresponding author at a reasonable request.

## Funding

This research was funded by the 10.13039/501100005147Key Project of Tackling Key Problems in Science and Technology of Henan Province under Grant No. 222102310066, and the Project of Basic Research Fund of Henan Institute of Medical and Pharmacological Sciences (No. 2023BP0204-3) & (No. 2022BP0119).

## Ethics declarations

This study was reviewed and approved by the Ethics Committee of Zhengzhou University, with the approval number: [ZZURIB 2019-001]. All participants/patients (or their proxies/legal guardians) provided informed consent to participate in the study.

## CRediT authorship contribution statement

**Keyan Wang:** Writing – review & editing, Writing – original draft, Validation, Investigation, Formal analysis, Data curation, Conceptualization. **Cuipeng Qiu:** Writing – review & editing, Visualization, Data curation. **Mengtao Xing:** Validation, Investigation, Formal analysis. **Miao Li:** Validation, Investigation. **Bofei Wang:** Investigation. **Hua Ye:** Supervision, Resources, Methodology. **Jianxiang Shi:** Resources, Methodology. **Liping Dai:** Supervision, Resources, Methodology. **Xiao Wang:** Writing – review & editing, Writing – original draft, Supervision, Project administration, Funding acquisition, Data curation, Conceptualization. **Peng Wang:** Writing – review & editing, Supervision, Resources, Project administration, Methodology.

## Declaration of competing interest

The authors declare that they have no known competing financial interests or personal relationships that could have appeared to influence the work reported in this paper.
