# Supplementary figures and images for: Association of elevated autoantibody to high expression of GNAS in hepatocellular carcinoma
Source: Heliyon. 2023 Nov 20;9(12):e22627. doi: 10.1016/j.heliyon.2023.e22627 (PMC10724561; doi:10.1016/j.heliyon.2023.e22627)

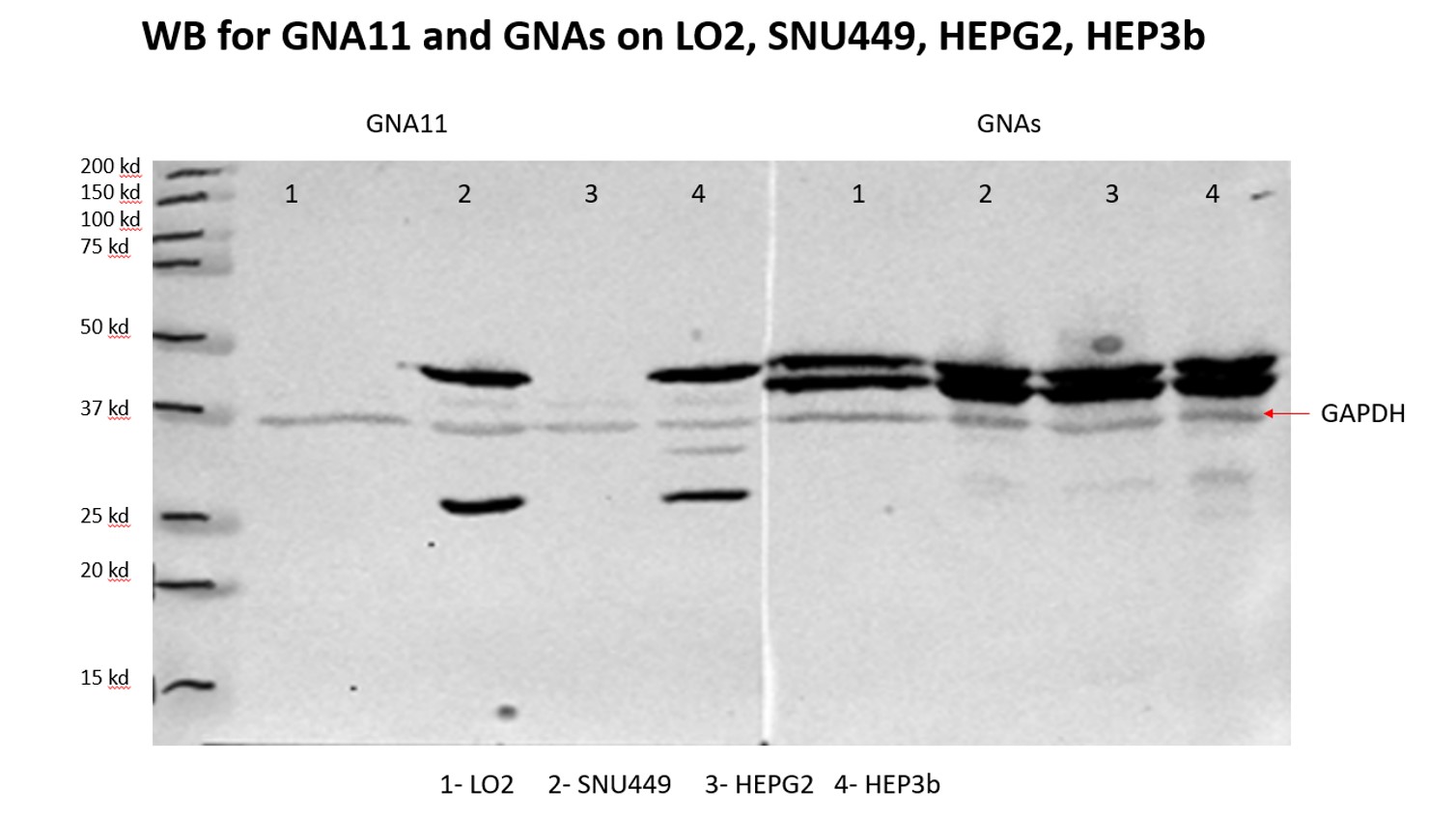


Supplement Figure 1 The uncropped versions of Figure 3B

Supplement: Multimedia component 1 [file mmc1.docx]
